# Ethnopharmacological study of medicinal plants used against skin ailments on Mount Pelion, central Greece

**DOI:** 10.3389/fphar.2023.1225580

**Published:** 2023-07-31

**Authors:** Efthymia Eleni Tsioutsiou, Antigoni Cheilari, Nektarios Aligiannis

**Affiliations:** Department of Pharmacognosy and Natural Products Chemistry, Faculty of Pharmacy, National and Kapodistrian University of Athens, Zografou, Greece

**Keywords:** ethnopharmacology, medicinal plants, skin ailments, mount Pelion, Greece

## Abstract

Throughout centuries, traditional herbal medicine and the employment of medicinal plants have constituted an important tool for the treatment and prevention of numerous diseases. The present study focuses on the collection of ethnopharmacological data regarding the uses of medicinal plants for the treatment of dermatological ailments in various villages of Mount Pelion, Greece. More specifically, the study area is represented by the city of Volos and villages located in Central West Pelion and has not been investigated up to now. The information on the medicinal uses of the various species was obtained through extensive semi-structured interviews or the completion of specific questionnaires by the informants. Although the Covid-19 pandemic caused difficulties and obstacles in carrying out this research procedure, 60 informants were recruited and interviewed (36 women and 24 men). Their age range was between 31 and 97 years and their educational level was characterized by great diversity (primary, secondary, and higher education). The elaboration of the gathered information included the calculation of some quantitative indices, such as Fidelity Level (FL), and Informant Consensus Factor (FIC). Moreover, the relative importance of each reported species was identified by calculating the Use Value (UV). The interviews revealed 38 plant taxa belonging to 27 plant families reported to be used in the study area exclusively against skin diseases. The plant family mostly mentioned by the informants was Hypericaceae, followed by Plantaginaceae and Amaryllidaceae, while among the most popular methods of application are cataplasms, compresses, and topical application of decoction or raw plant material. Some of the most cited species are *Hypericum perforatum* L., *Quercus coccifera* L., and *Plantago* sp., traditionally used to treat skin problems such as eczema, wounds, and insect stings. The present ethnopharmacological study is the first documentation of ethnobotanical knowledge of this area that points out the traditional uses of medicinal plants against skin ailments.

## 1 Introduction

Ever since the development of mankind and advanced civilizations, the healing activities of a great number of medicinal plants were evidenced, reported, and communicated to successive generations, highlighting the importance of the dissemination of ethnopharmacological knowledge (Petrovska, 2012). The uses of plant species in folk medicine demonstrate the strong connection between human communities and nature and constitute a cultural heritage that tends to vanish due to socio-economic and land use changes (Danna et al., 2022). In Greece, medicinal plants’ use to treat several illnesses including skin diseases dates back to ancient times, when Hippocrates (fifth century BC) and Dioscorides (first century AD) established the scientific aspect of medicine based on the healing properties of different plant species (Hanlidou et al., 2004). In Greek mythology, Cheiron was a centaur renowned for his skills in prophecy, astrology, botany, pharmacy, and mainly in the science of herbs and medicine. The forested slopes of Pelion are where, according to tradition, Cheiron the centaur practiced the art of healing with herbs (Lietava, 1992). The traditional knowledge was preserved through centuries in the study area and represents an important pillar not only of traditional cultural and folkloristic heritage, but also of Greek traditional medicine. The Mediterranean basin is one of the richest biodiversity hotspots due to its intricate topographical, geographical, and climatic factors (Kougioumoutzis et al., 2021). Greece hosts 7,043 native plant taxa, 1435 of which are Greek endemics (Dimopoulos et al., 2016). In Greece, the number of ethnobotanical studies on traditional uses of medicinal plants is scarce. The recent surveys concerning the knowledge of medicinal plants of Greece were carried out in the regions of Zagori (Malamas and Marselos, 1992; Vokou et al., 1993), Thessaloniki (Kleftoyanni and Kokkini, 2003; Hanlidou et al., 2004; Karousou et al., 2007), Crete (Skoula et al., 2009), Mt. Pelion (Brussell, 2004), Greek Islands of North Aegean (Axiotis et al., 2018), Central Macedonia (Tsioutsiou et al., 2019), Lemnos island (Papageorgiou et al., 2020), Peloponnesus (Petrakou et al., 2020), and more recently on Milos island (Cyclades) (Perouli and Bareka, 2022). More specifically, near the study area only one ethnobotanical study was conducted in the past, including general information on the traditional uses of plants, but not exclusively for medicinal purposes. Even though very few ethnobotanical studies have been conducted in Greece, none of them was exclusively focused on the use of plant species against a specific category of pathological conditions such as skin diseases, that represent one of the most common categories of ailments in the history of medicine. Nowadays, skin disorders are a public health problem in many parts of the world, while dermatological disease treatment is a global concern, especially in the case of chronic wounds, where despite scientific progress their comprehensive treatment remains still a challenge (Posnett et al., 2009). Skin diseases are numerous and harmful in many ways, and they affect people of all ages from neonates to the elderly. Some skin pathological conditions such as eczema, wounds, psoriasis, and impetigo are among the top 50 most prevalent diseases globally. Moreover, skin diseases are the fourth leading cause of the non-fatal disease burden, highlighting the need for finding ways to manage them (Seth et al., 2017). Considering the scarcity of published ethnopharmacological information along with the significant floristic diversity of the region of Thessaly and in particular of Mount Pelion, our aim is to survey medicinal plants and their traditional uses for the treatment of different skin diseases, which are undoubtfully a common health difficulty. Finally, this is the first quantitative ethnomedicinal study of therapeutic herbs utilized against skin diseases intended for the discovery of bioactive natural products for their treatment, as well as their inclusion in future global strategies, in order to improve the health of the affected populations worldwide (Hay et al., 2014).

## 2 Materials and methods

### 2.1 Study area

**FIGURE 1:**
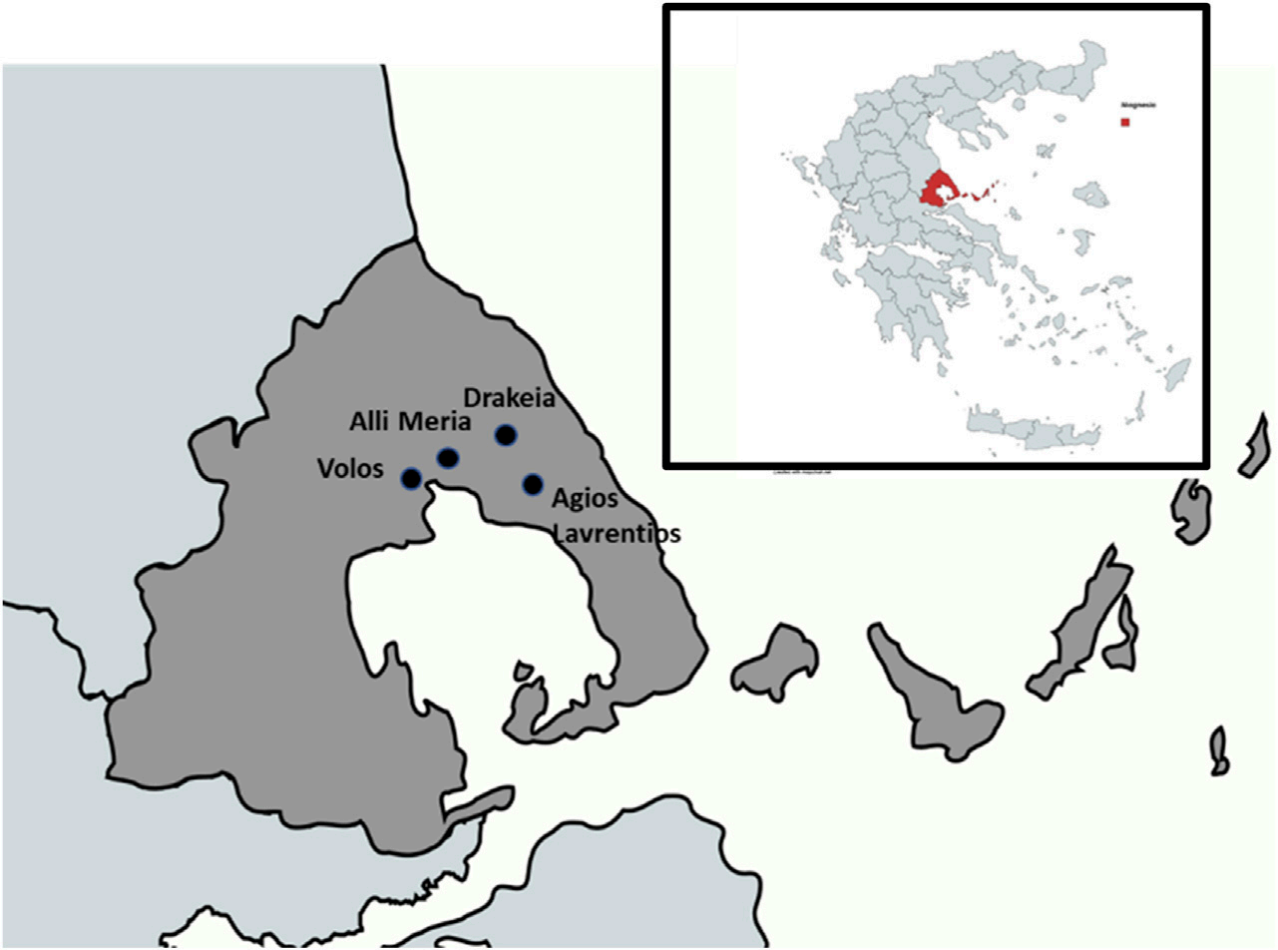
Map of Volos and the nearby villages of the study area.

The study was conducted on Mount Pelion, which forms a hook-like peninsula between the Pagasetic Gulf and the Aegean Sea and is located in the southeastern part of Thessaly in central Greece. Its foothills extend along the Magnesia Peninsula in the eastern seaboard of the city of Volos. Volos is a coastal city in the Thessaly region with a population of about 125,000 (Papanastasiou and Melas, 2009). The city is located in an area of complex topography on the northern side of the Pagasetic Gulf, on the east coast of central Greece and its climate is of Mediterranean type with wet mild winters and hot dry summers (Moustris et al., 2016). The area where the study was carried out includes the Central West Pelion and more specifically the villages Agios Lavrentios, Drakeia, Agios Vlasios, and Alli Meria (Figure 1). Around 80% of the informants were inhabitants of the village Agios Lavrentios. Central West Pelion was chosen for the present ethnobotanical investigation because it has been poorly studied to date, as only one ethnopharmacological survey has been effectuated in Mount Pelion area (Brussell, 2004). It represents an area of special interest due to the survival of cultural patrimony, such as several ancient traditions and festivities. For example, a representative custom in the village of Makrinitsa, and other villages of Central West Pelion, is called “Maides” (Greek: Μάηδες), and is celebrated on the first of May. During this feast, the villagers celebrate the annual rebirth of spring and the fertility of plants, soil, and animals through dances and reenactments (Chourmouziadis et al., 1982). As mentioned above, most of the interviews took place in Agios Lavrentios, which is a village amphitheatrically built at an altitude of 600 m on the slopes of the mountain and 22 km east of Volos. Nowadays the population reaches 180 inhabitants, whereas in the early 20th century and up to 1930 amounted to 2000 people (Vamvakos, 1927). The majority of the informants in all four villages are farmers and their main work field is tree cultivation (olives, apples, pears, chestnuts, cherries) (Niavis et al., 2018). The construction of Agios Lavrentios village begins with the establishment of the Monastery of Saint Lawrence by Benedictine monks from Amalfi, Italy. The Monastery was re-established in 1378 by Saint Lawrence monk of the Monastery of the Great Lavra of Mount Athos. In 1389, after the battle of Kosovo, the invasion of Ottomans in the Balkans led to important population movements. Vlachs and Arvanites settled in the area and the first residential core was constructed in Servanates, a settlement situated below the current village. In 1550, the inhabitants of Servanates started moving to the northern part and built the village which exists thus far (Papathanasiou, 2006).

### 2.2 Methodology

The fieldwork was conducted during the autumn of 2020 and the summer of 2021. The ethnopharmacological data regarding the use of herbal preparations, exclusively against skin disorders, were gathered through extensive semi-structured interviews with the informants, which often led to open discussions. The interviews were recorded in the houses of the local people or squares of the villages respecting all the safety restrictions imposed by the COVID-19 pandemic. During the interviews also a questionnaire was distributed and completed by the local people aiming at an efficient organization of the data. It is important to underline that the social impact of the pandemic aggravated the research procedure. Though, 60 informants were recruited and interviewed. For every informant, personal information about age, gender, education level, and profession was recorded and facilitated the analysis and comparison of the ethnobotanical knowledge in relation to these factors. During the interviews, the informants were requested to indicate vernacular or botanical names of species, parts of the plant used, association with other plants, traditional ethnopharmacological uses against skin disorders, and preparation procedures. In certain cases, information on specific recipes and their ingredients as well as on the method of remedies conservation was included. In addition, the informants were asked to define the sources from which the species are procured (collected by themselves, collected by other people, herbal shops, internet), the sources from which the reported information originates (other people of the community, herb sellers, books or seminars), while they were also requested to provide information on the endemism of the listed species. The practical difficulties and the restricted sanitary circumstances attributed to the COVID-19 pandemic prevented us from visiting the fields together with the informants as well as from collecting and vouchering the reported species. In contemplation of facilitating the species identification and enriching the variety of data, images of the plants were indicated to the informants, to help them both recognize the plant species and remind eventual medicinal plants used in the past against skin diseases. Many of the informants had personal experience in using and recognizing medicinal plants, as a consequence of the transmission of ethnobotanical knowledge through generations. Nevertheless, the identification procedure involved the aforementioned limitations and since it was mainly performed through photographs and not via specimens, in cases of doubt we referred only to the genera, while the species identification was avoided (*Thymus* sp., *Plantago* sp., *Taraxacum* sp.). In the case of *Plantago* sp. it was reported that *Plantago lanceolata* L., *Plantago major* L. and *Plantago media* L. were used by the informants in the same ways. Although the species identification was not successfully performed, all three species are part of the local flora. In case of *Thymus* sp. the most probable species to which the genus refers is *Thymus capitatus* Hoffmanns. and Link, that is a synonym of the accepted name *Thymbra capitata* (L.) Cav representing a very common species in the flora of Mount Pelion (Flora of Greece web Vascular Plants of Greece An annotated Checklist, 2023). In regard to *Taraxacum* sp. the most likely species identification is *Taraxacum officinale* F.H. Wigg. subsp. *officinale*, a synonym of the accepted name *Taraxacum* sect. Ruderalia Kirschner and al. All information was obtained after receiving oral and written consent from the participants, according to the ISE (International Society of Ethnobiology) Code of Ethics. The botanical names and families of the reported species were confirmed through the databases “World Flora Online Plant List” and “The Global Biodiversity Information Facility” (Global Biodiversity Information Facility, 2022; World Flora Online Plant List, 2022). The data concerning the plant uses reported, were organized using Microsoft Excel. The skin ailments cited were summarized and classified into 18 different categories based on the characteristics of the symptoms and their relief. Every citation of each medicinal use was represented by a single row and the citations were evaluated and statistically elaborated relying on the categorization of the ailments. Several ethnobotanical indices were adopted for the analysis and evaluation of the collected data and were calculated using the classification of skin ailments as a point of reference. An interesting comparison was also effectuated contrasting the data collected through the conduction of the present ethnopharmacological research and the data reported in the total of ethnobotanical or ethnopharmacological studies carried out in Greece. For this purpose, we consulted our recent review (Tsioutsiou et al., 2022), which indicates the medicinal plants used traditionally for skin related problems in the south Balkan and east Mediterranean region. Moreover, the ethnopharmacological background of the study area was accentuated by comparing the obtained information to the already existing evidence on the local uses of medicinal plants against skin diseases described in the study of Brussell. (2004), which represents the only published literature relevant to this topic.

### 2.3 Quantitative ethnobotanical indices

#### 2.3.1 Informant Consensus Factor (FIC)

The Informant Consensus Factor (FIC) (Trotter and Logan, 1986) was calculated for each ailment category, aiming to evaluate the uniformity of the ethnobotanical knowledge and estimate the consensus among the informants on the use of medicinal plants for skin disease categories. The FIC was calculated using the following formula:

$$FIC=\frac{Nur-Nt}{Nur-1}$$

where Nur refers to the number of citations used in each ailment category and Nt is the number of taxa used in the same ailment category (Singh et al., 2012). The values of this index range from 0 to 1. A high FIC value reflects an agreement of the informants on the use of taxa for a specific category of disease, whereas a low FIC value indicates heterogeneity of information provided by different informants and a low exchange of traditional knowledge on the use of medicinal plants in the community (Heinrich, 2000).

#### 2.3.2 Fidelity level (FL)

The Fidelity Level index (FL) of each plant is expressed as the percentage of the ratio between the number of informants who suggested the use of a specific ailment category (Ip) and the total number of informants who mentioned the plant for any use (Iu) (Friedman et al., 1986). It was calculated by the following formula:

$$FL\%=\frac{Ip}{Iu}\times 100$$

In the present study, the FL index was measured for the first 12 most cited species which were mentioned by at least 8 informants for all ailment categories.

#### 2.3.3 Use Value (UV)

The Use Value (UV) is an ethnobotanical index widely used to quantify the relative importance of useful plants. It is mostly applied to indicate noteworthy species and it combines the frequency of a species citation with the number of uses mentioned per species (Zenderland et al., 2019). UV is an index introduced by Phillips et al. (1994) and simplified by Rossato and Leitao-Filho (1999). It was calculated using the following formula:

$$UV=\frac{Nr}{Ni}$$

where Nr is the total number of medicinal use citations in all therapeutic categories for all informants, and Ni is the total number of informants (Doyle et al., 2017).

## 3 Results

The interviews revealed that in Central West Pelion medicinal plants along with traditional herbal practices are commonly employed against skin ailments. Some frequent skin problematic conditions mentioned by the interviewed inhabitants are wounds, cuts, burns, eczema, and psoriasis. The skin ailments cited in the present study were summarized and classified into 18 different categories based on the characteristics of the symptoms and their relief (Table 1). As mentioned above, the elaboration of the data included the evaluation of some quantitative indices using the classification of skin ailments as a point of reference for the report of every citation. These indices are the Informant Consensus Factor (FIC) (Table 1), and the Fidelity Level (FL) (Table 2). Moreover, the relative importance of each reported species was identified by calculating the Use Value (UV) (Table 3).

**TABLE 1:** Skin disease categories and informant consensus factor (FIC).

Skin disease	Number of citations (Nur)	Number of taxa (Nt)	FIC	Ailment category
Acne	3	3	0	C1
Alopecia, Trichotillomania	18	6	0.71	C2
Anti-inflammatory, Analgesic	12	5	0.64	C3
Aphthae, Mouth sores	28	2	0.96	C4
Bruises, Contusions, Oedemas	40	8	0.82	C5
Calluses	11	2	0.90	C6
Cuts, Wounds, Burns, Ulcers	114	14	0.88	C7
Eczema	18	2	0.91	C8
Freckles	2	1	1.00	C9
Furuncles, Boils, Spots	20	3	0.89	C10
Haemorrhoids	9	4	0.63	C11
Haemostatic	3	1	1.00	C12
Herpes	1	1	0	C13
Insect stings, Animal bites	36	6	0.86	C14
Irritations	14	2	0.92	C15
Psoriasis	21	3	0.90	C16
Skin and eye antiseptic, Antifungal	14	5	0.69	C17
Warts	1	1	0	C18

**TABLE 2:** Fidelity Level Index (FL) for the most relevant species (≥8 citations).

	Number of informants citing for all ailment categories	C1	C2	C3	C4	C5	C6	C7	C8	C9	C10	C11	C12	C13	C14	C15	C16	C17	C18
*Allium cepa* L.	11			9		91		55			18								
*Allium sativum* L.	10	10		70		70									20			10	10
*Fraxinus ornus* L.	10							100					30						
*Hylotelephium spectabile* (Boreau) H. Ohba	10						100												
*Hypericum perforatum* L.	47	2				21		100						2	2		21		
*Malva sylvestris* L.	12														83	83			
*Matricaria chamomilla* L.	11	9			64			36								27		73	
*Morus nigra* L.	21				100														
*Plantago* sp.	20					10		100			80				95			5	
*Prunus avium* (L.) L.	8							100											
*Quercus cerris* L.	8							63	25								25		
*Quercus coccifera* L.	16								100								56		

**TABLE 3:** Ethnopharmacological data on the uses of plant species against skin ailments.

Botanical name, family name	Local name	Plant part (s) used	Preparation	Utilization method	Ailment treated	UV	Wild (W) or cultivated (C)/Native (N) or imported (I)	Ethnobotanical reports in Greece Tsioutsiou et al. (2022)
*Aesculus hippocastanum* L., Sapindaceae	Pikrokastania	Fruits	Melted with petroleum	Topically applied	Odemas (2)	0.07	W/N	+
		Fruits (powder)	Immersed in oil	Topically applied	Oedemas (1), Haemorrhoids (1)			
*Allium cepa* L., Amaryllidaceae	Kremmidi	Bulbs	Heated and/or mixed with wine	Cataplasm	Bruises/contusions/oedema (10), Furuncles/spots (2), Wounds (6)	0.32	C/I	+
		Bulbs	Fresh	Cataplasm	Nail inflammation (1)			
*Allium sativum* L., Amaryllidaceae	Skordo	Bulbs	Crushed and mixed with soap, “tsipouro” and egg white	Cataplasm	Oedema (7), Skin inflammation (7)	0.32	C/I	+
		Bulbs	Smashed and mixed with vinegar	Topically applied	Insect bites (2)			
		Bulbs	Fresh	Topically applied (rubbed)	Acne (1), Fungal dermatosis (1), Warts (1)			
*Aloe vera* (L.) Burm.f., Asphodelaceae	Aloi	Leaves	Fresh	Topically applied	Cuts/wounds/burns (1)	0.02	C/I	+
*Anemone coronaria* L., Ranunculaceae	Anemoni	Bulbs	Crushed and mixed with flour	Eaten	Haemorrhoids (4)	0.07	W/N	
*Artemisia absinthium* L., Asteraceae	Apsithia	Aerial parts	Decoction	Topically applied	Wounds (3)	0.05	W/N	+
*Asphodelus fistulosus* L., Asphodelaceae	Sferdoukli, Agiokremmida	Bulbs	Fresh	Topically applied (rubbed)	Alopecia (3), Fungal dermatosis (lichens) (1)	0.07	W/N	
*Asplenium ceterach* L., Aspleniaceae	Skorpidi	Leaves	Fresh	Cataplasm	Bruises/contusions (6), Wounds (1)	0.12	W/N	
*Citrus* x *limon* (L.) Osbeck., Rutaceae	Lemoni	Fruits	Fruits’ juice	Cataplasm	Skin inflammation (1), Oedema (1)	0.03	C/I	
*Clematis flammula* L., Ranunculaceae	Agrabeli	Leaves	Fresh	Topically applied (rubbed)	Trichotillomania (6)	0.10	W/N	
*Dioscorea communis* (L.) Caddick & Wilkin, Dioscoreaceae	Vergia	Fruits and rhizomes	Immersed in olive oil	Oil topically applied	Skin and muscular pain (2)	0.03	W/N	+
*Equisetum arvense* L., Equisetaceae	Politrichi, Ekuizeto, Komi Afroditis	Aerial parts, whole plant	Decoction	Washes	Alopecia (4)	0.07	W/N	+
*Ficus carica* L., Moraceae	Siko	Leaves, stems	Fresh, juice	Topically applied	Bee stings (3), Irritations (caused by *U. dioica*) (1)	0.07	W/N	+
*Foeniculum vulgare* Mill., Apiaceae	Agiomarathos	Aerial parts	Crushed	Topically applied	Wounds/cuts (2)	0.03	W/N	+
*Fraxinus ornus* L., Oleaceae	Fraxos, Thrapso	Flowers, stems’ bark	Fresh	Topically applied	Wounds/ulcers (6) Haemostatic (3)	0.22	W/N	+
		Flowers, stems	Immersed in olive oil	Topically applied	Cuts/wounds/burns (4)			
*Hedera helix* L., Araliaceae	Bruskli, Kissos	Leaves	Mixed with oil and sugar	Cataplasm	Furuncles/boils (2)	0.03	W/N	+
*Hylotelephium spectabile* (Boreau) H. Ohba, Crassulaceae	Gatos	Leaves	Fresh, leaves’ juice	Topically applied	Calluses (10)	0.17	C/I	
*Hypericum perforatum* L., Hypericaceae	Valsamochorto, Valsamo	Aerial parts	Immersed in olive oil	Oil topically applied	Wounds/cuts/burns (16)	1.17	W/N	+
		Inflorescences	Immersed in olive oil	Oil topically applied	Oedema (10), Psoriasis (10), Wounds/cuts/burns (27)			
		Flowers	Mixed with wax	Cataplasm	Wounds (1)Herpes (1)			
		Whole plant	Mixed with milk or immersed in olive oil	Cataplasm	Acne (1) Cuts/wounds/burns (1)			
		Whole plant	Immersed in olive oil	Oil topically applied	Insect stings (1), Wounds/cuts/burns (2)			
*Laurus nobilis* L., Lauraceae	Dafni	Seeds	Decoction	Washes	Alopecia (3)	0.05	W/N	+
*Malva sylvestris* L., Malvaceae	Molocha	Leaves	Fresh	Topically applied (rubbed)	Insect stings (10), Irritations (caused by *Urtica dioica* L.) (10)	0.33	W/N	+
*Matricaria chamomilla* L., Asteraceae	Chamomili	Flowers	Decoction	Gargles	Aphthae/mouth sores (3)	0.38	W/N	+
		Flowers	Decoction	Compress	Eye inflammation (3), Aphthae/mouth sores/antiseptic (4), Fungal dermatosis (2), Burns (4)			
		Inflorescences	Fresh	Topically applied	Antiseptic (2), Irritations (2)			
		Whole plant	Decoction	Compress	Acne (1), Antiseptic/eye inflammation (1), Irritations (1)			
*Melissa officinalis* L., Lamiaceae	Melissochorto	Leaves	Fresh	Topically applied	Insect stings (1)	0.02	W/N	+
*Morus nigra* L., Moraceae	Muro	Fruits	Fruit juice boiled with sugar	Topically applied	Aphthae/mouth sores (21)	0.35	W/I	
*Nicotiana tabacum* L., Solanaceae	Kapnos	Leaves	Crushed	Topically applied	Wounds (1)	0.02	C/I	+
*Olea europaea* L., Oleaceae	Elia	Fruit	Oil mixed with whitewash	Topically applied	Wounds (1)	0.02	C/N	+
*Parietaria officinalis* L., Urticaceae	Perdikaki, Gri chorto	Stems	Boiled in red wine	Cataplasm	Wounds (1)	0.03	W/N	+
		Leaves	Mixed with penicillin	Topically applied	Wounds (1)			
*Plantago* sp., Plantaginaceae	Pedanevro	Leaves	Fresh	Cataplasm, topically applied	Wounds (20), Insect stings/animal bites (19), Furuncles (16), Antiseptic (1), Bruises (3)	0.97	W/N	+
*Prunus avium* (L.) L., Rosaceae	Kerasi	Leaves	Immersed in wine, mixed with tomato pulp	Cataplasm	Wounds/burns (8)	0.13	C/N	
*Quercus cerris* L., Fagaceae	Pournari	Rhizomes (external part)	Decoction	Topically applied	Wounds (5), Eczema (2), Psoriasis (2)	0.15	W/N	
*Quercus coccifera* L., Fagaceae	Pournari	Rhizomes (external part)	Decoction	Topically applied, affected area exposed to the vapors of decoction	Eczema (16), Psoriasis (9)	0.42	W/N	
*Sambucus nigra* L., Viburnaceae	Sabukos	Inflorescences	Decoction	Topically applied	Wounds (2), Haemorrhoids (3)	0.10	W/N	+
		Stems’ bark	Heated with olive oil	Cataplasm	Burns (1)			
*Sedum urvillei* DC., Crassulaceae	Amarados	Leaves	Fresh	Cataplasm	Calluses (1)	0.02	W/N	
*Sinapis arvensis* L., Brassicaceae	Sinapi	Seeds	Decoction	Cataplasm	Skin inflammation (1), Oedema (1)	0.03	W/N	
*Solanum melongena* L., Solanaceae	Melitzana	Fruits	Cooked and powdered	Topically applied	Haemorrhoids (1)	0.02	C/I	
*Taraxacum* sp., Asteraceae	Pikralida	Leaves	Boiled	Topically applied	Skin antiseptic (3)	0.05	W/N	
*Thymus* sp., Lamiaceae	Thymari	Flowers	Essential oil	Topically applied	Alopecia (1)	0.02	W/N	
*Urtica dioica* L., Urticaceae	Tsouknida	Aerial parts	Decoction	Washes	Alopecia (1)	0.02	W/N	
*Vitis vinifera* L., Vitaceae	Klima	Bark	Juice of bark	Topically applied	Freckles (2)	0.03	C/N	+

### 3.1 Demography of informants

A total of 60 informants were interviewed. Out of these 36 were female (60%) and 24 were male (40%). Their age range is between 31 and 97 years and their educational levels include primary, secondary, and higher education. In more detail, the age of 13 female informants ranged from 30 to 50 years, 12 were between 50 and 70 years, and 11 female informants were older than 70 years. On the other hand, the age of 11 male informants ranged from 30 to 50 years, 8 were between 50 and 70, and 5 informants were older than 70 years. It is noteworthy that 59 out of 60 informants indicated that information on medicinal plant uses emanates from individuals of the community, as friends and family, while only 2 mentioned accessing ethnopharmacological information through herb sellers, and only 3 communicated that their knowledge derives from books and seminars. This fact highlights the importance of traditional ethnobotanical knowledge that passes down through generations, as well as the substantial necessity to carry out more ethnobotanical and ethnopharmacological surveys. In respect of the medicinal plants source the majority of the informants, 38 out of 60, declared to use species collected by other people in their village or nearby villages, 21 of them collect herbs by themselves, and only 2 buy the species from various shops. This demonstrates that the provincial societies of Greece are self-sufficient, and people have personal experience in self-medication using herbs.

### 3.2 Most cited families and species

**FIGURE 2:**
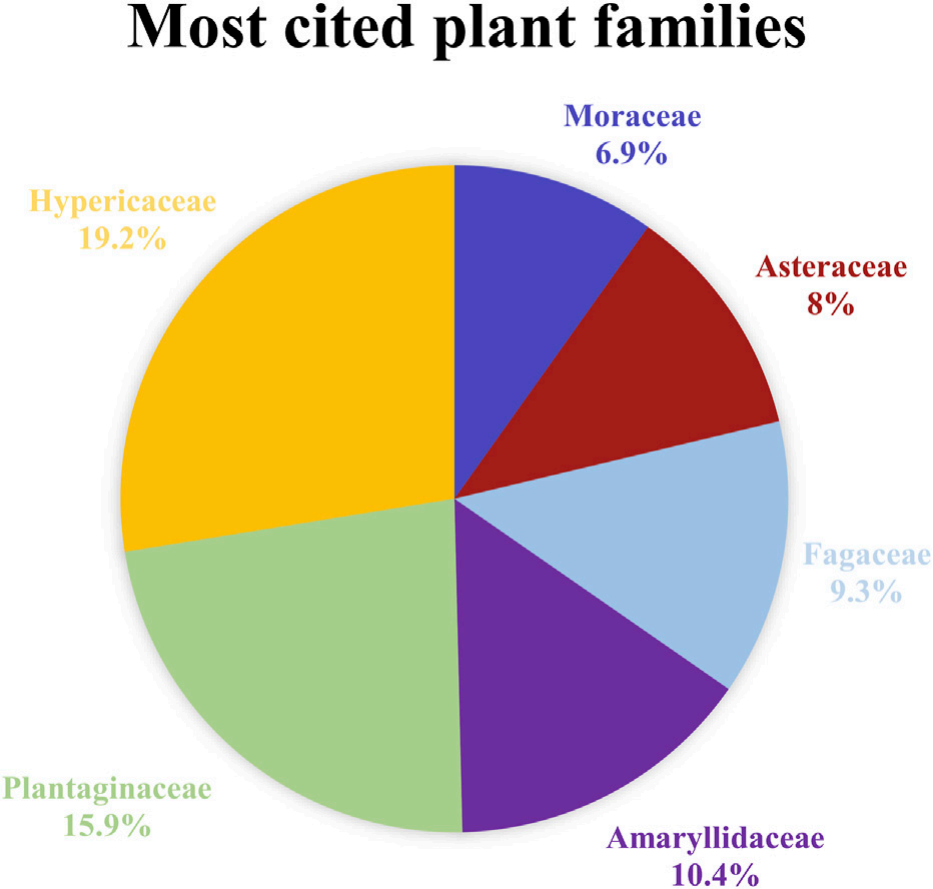
Most cited plant families.

**FIGURE 3:**
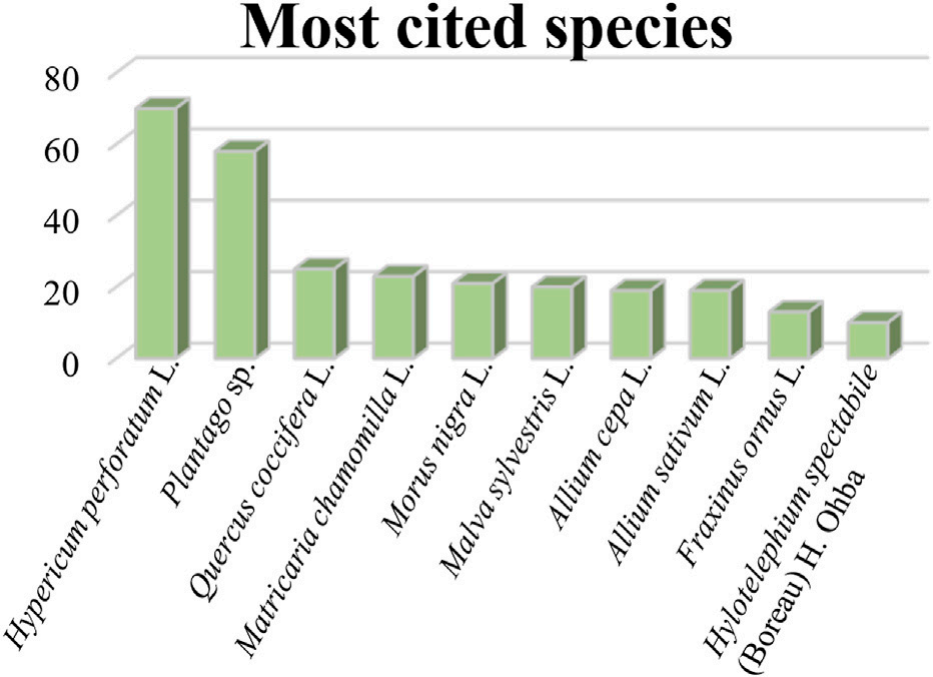
Most cited plant species.

The informants reported 38 plant species belonging to 27 plant families. The most represented families (Figure 2) are Hypericaceae (70 citations, 19.2%), Plantaginaceae (58 citations, 15.9%), Amaryllidaceae (38 citations, 10.4%), Fagaceae (34 citations, 9.3%), followed by Asteraceae (29 citations, 8%), Moraceae (25 citations, 6.9%), Malvaceae (20 citations, 5.5%), Oleaceae (14 citations, 3.8%), Crassulaceae (11 citations, 3%), Ranunculaceae (10 citations, 2.7%), Rosaceae (8 citations, 2.2%), Aspleniaceae (7 citations, 1.9%), Viburnaceae (6 citations, 1.6%), Asphodelaceae (5 citations, 1.4%), Sapindaceae and Equisetaceae (4 citations, 1.1%), Lauraceae and Urticaceae (3 citations, 0.8%), Apiaceae, Araliaceae, Brassicaceae, Dioscoreaceae, Lamiaceae, Rutaceae, Solanaceae, Vitaceae (2 citations, 0.6%). The surveyed species are listed in alphabetical order in Table 3, where for each taxon cited, data on scientific name, family, local name, part of the plant used, preparation, medicinal use, and UV index are documented. The 10 most cited medicinal plants (Figure 3) are *Hypericum perforatum* L. (70 citations), *Plantago* sp. (58 citations), *Quercus coccifera* L. (25 citations), *Matricaria chamomilla* L. (23 citations), *Morus nigra* L. (21 citations), *Malva sylvestris* L. (20 citations), *Allium cepa* L. (19 citations), *Allium sativum* L. (19 citations), *Fraxinus ornus* L. (13 citations), *Hylotelephium spectabile* (Boreau) H. Ohba, (10 citations). These species were reported to be used for more than one ailment category, apart from *H. spectabile* and *M. nigra*, which were mentioned to be used only for one category of skin disease. The juice of the leaves of *H. spectabile* is topically applied on the skin for its keratolytic activity against calluses, and *M. nigra* is traditionally applied in the oral cavity for the cure of aphthae and mouth sores. In Table 3, it is also signed if the species is wild or cultivated, native or imported and if it has been previously cited in any other ethnobotanical study conducted in Greece for the treatment of skin ailments. This comparison showed that 61% of the species cited in the present study have been also mentioned to be employed against skin diseases in other ethnobotanical studies carried out in Greece up to now. However, it is crucial to emphasize the novelty of ethnopharmacological information, represented by 39% of the species cited in the present study for the treatment of skin ailments, that have not been recorded in any ethnobotanical study conducted in Greece in the past for the same purpose. These species are Q. *coccifera*, *Q. cerris* L., *M. nigra*, *Anemone coronaria* L., *H. spectabile*, *Prunus avium* (L.) L., *Clematis flammula* L., *Asplenium ceterach* L., *Asphodelus fistulosus* L., *Citrus* x *limon* (L.) Osbeck., *Sedum urvillei* DC., *Sinapis arvensis* L., and *Solanum melongena* L. Besides, *Taraxacum* sp., and *Thymus* sp. are not mentioned as identified species in previous ethnobotanical studies carried out in Greece, but their genus is referred to the plant list without specifying the species. The estimate of similarities and dissimilarities between our study and the ethnobotanical study conducted in the past by Brussel in the same area led to interesting observations. Although the reports presented in Brussel’s ethnobotanical survey refer to different ailment categories, we focused on the data relative to skin diseases. The plant species used against skin ailments in both studies are *Hedera helix* L., *H*. *perforatum*, *M. chamomilla*, *Plantago* sp., *Parietaria officinalis* L. In Brussel’s study *H. helix* leaves are used to prepare a decoction employed against skin ulcerations and rashes and as hair tonic, while in the present study it is described that leaves are mixed with oil and sugar and applied as cataplasm to treat boils and furuncles. In our study *H. perforatum* aerial parts and inflorescences are used in different preparation forms for the treatment of wounds, cuts, burns, psoriasis, oedema, and acne, while in the past study it was cited to be used for the cure of cuts, as a poultice prepared out of leaves and stems. *M. chamomilla* decoction is commonly mentioned to be applied as compress to heal eye inflammation. The inhabitants of the study area indicated that the stems of *P. officinalis* boiled in red wine are administered as cataplasm for the treatment of wounds. On the other hand, in the study conducted by Brussel, the juice of leaves and stems was applied topically against cuts and bruises. Finally, *Plantago* sp. leaves are reported to be efficient as cataplasm in case of wounds, insect stings, animal bites, furuncles, bruises and as antiseptic. In the previous records the species was specified while the inhabitants declared to utilize leaves of *P. lanceolata* to prepare a decoction against acne and eye inflammation. However, a few common uses against skin ailments were outlined, various plant species cited in the present study were also assessed in the previous ethnobotanical study for the treatment of other categories of diseases. These are *Artemisia absinthium* L., *A. ceterach*, *Sambucus nigra* L., *Foeniculum vulgare* Mill., *Urtica dioica* L., *Vitis vinifera* L., *Equisetum arvense* L., *Q. coccifera*, *Aesculus hippocastanum* L., *Melissa officinalis* L., *M. sylvestris*, *Ficus carica* L., and *F. ornus*.

### 3.3 Plant parts used

**FIGURE 4:**
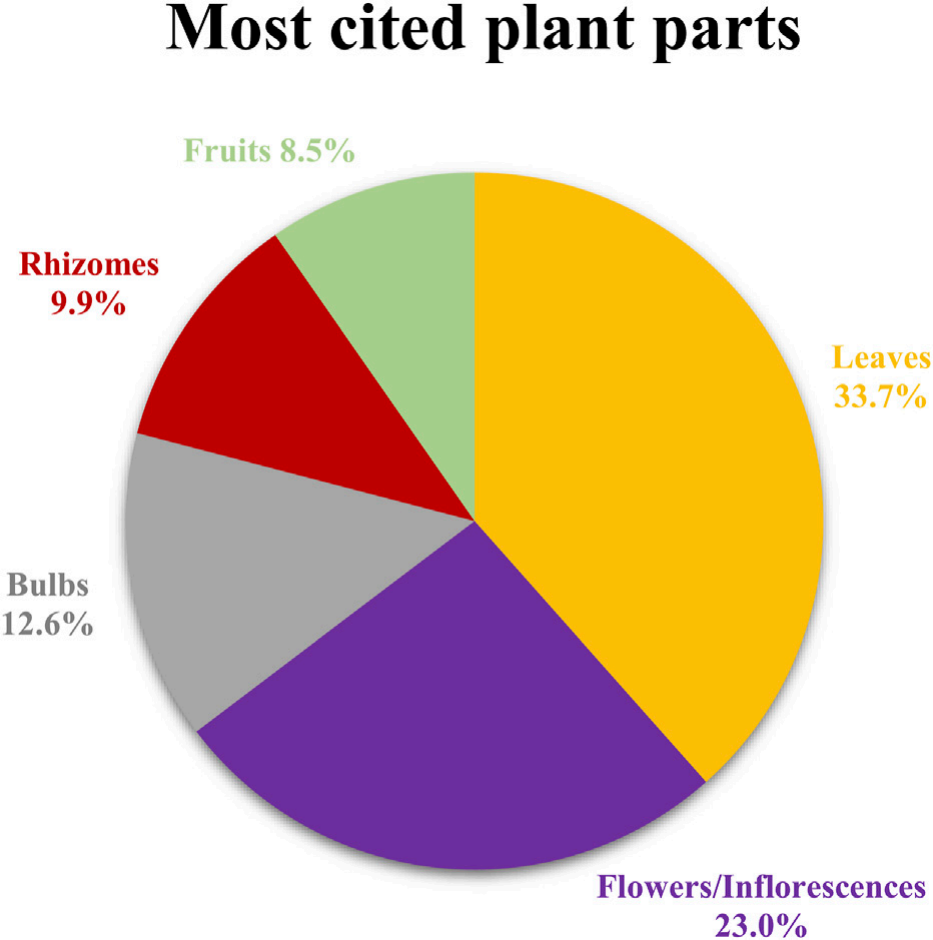
Most cited plant parts.

The plant part most frequently used either directly or to prepare the remedies against skin ailments (Figure 4) are leaves (123 reports, 33.7%). However, also other plant parts were indicated: flowers/inflorescences (84 reports, 23%), bulbs (46 reports, 12.6%), rhizomes (36 reports, 9.9%), fruits (31 reports, 8.5%), aerial parts (23 reports, 6.3%), stems (13 reports, 3.6%), whole plant (11 reports, 3%), seeds (5 reports, 1.4%), bark (2 reports, 0.6%).

### 3.4 Preparation forms

**FIGURE 5:**
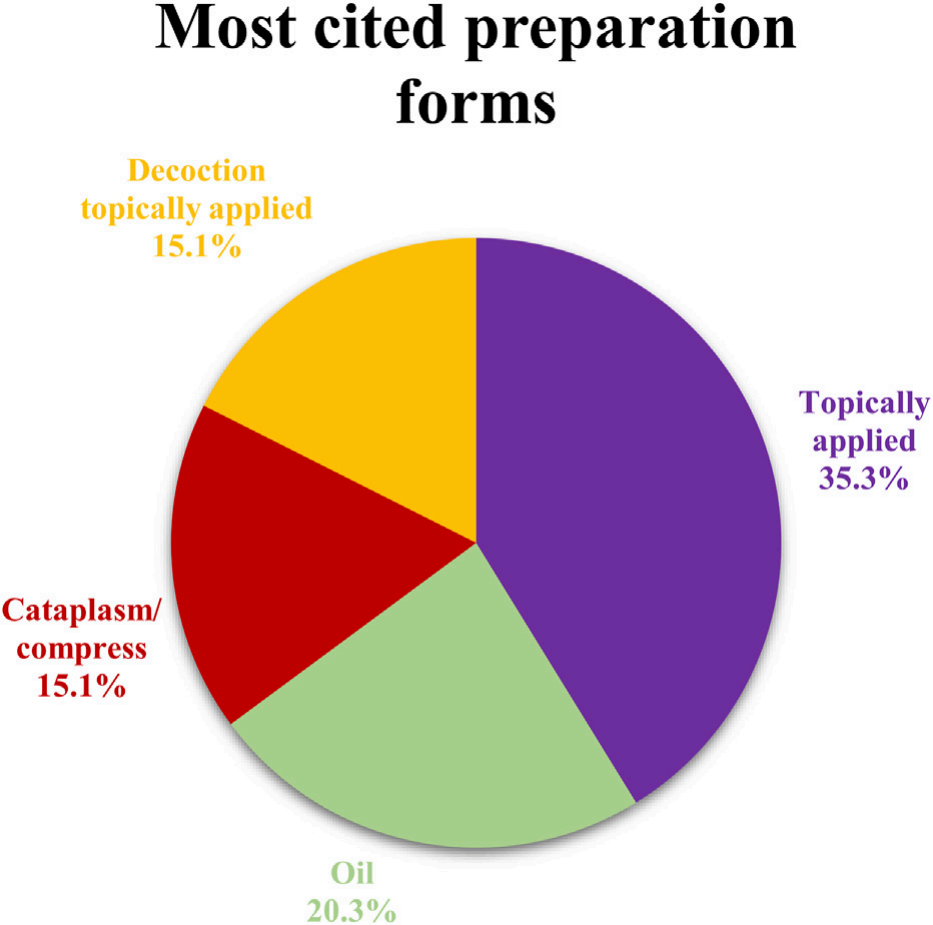
Most cited preparation forms.

**FIGURE 6:**
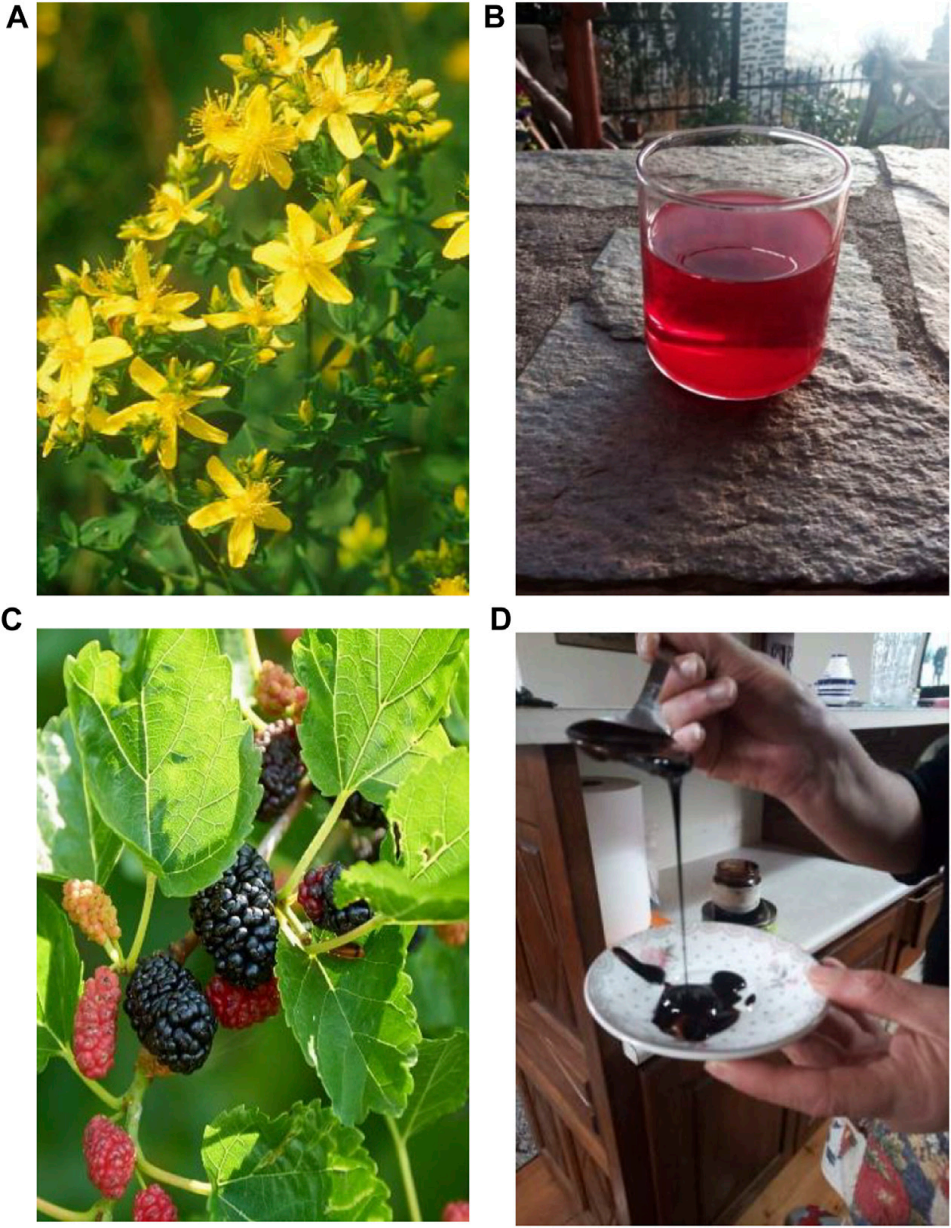
Plant species and preparations commonly used in the study area for the treatment of skin diseases: **(A)** *H. perforatum* inflorescences, **(B)** Oil prepared from *H. perforatum* inflorescences, **(C)** *M. nigra* fruits, **(D)** Solution prepared from *M. nigra* fruits.

The preparation forms as well as the methods of application of the different herbal medicines constitute one of the most considerable aspects of an ethnopharmacological survey. In this study, the information collected was exclusive to the use of plants for the treatment of skin disorders. This justifies the fact that the most prevalent method of administration is the external or topical application of the different preparation forms. The most frequent method is the topical application of the fresh plant part on the skin (129 citations, 35.3%). A characteristic example is the leaves of *Plantago* sp. for the treatment of cuts, wounds, and burns. Oils and more precisely oily extracts of species are reported to be used against various skin problems (74 citations, 20.3%). Cataplasms compresses and decoctions topically applied are often and equally employed (55 citations, 15.1% each). A notable percentage of informants cited topical rubbing of species on the affected area (27 citations, 7.4%). Other preparation forms are decoctions applied as washes, cataplasms, and gargles (8 citations, 2.2%, 5 citations, 1.4%, and 3 citations, 0.8% respectively). Finally, in some rare cases, the plant part is eaten, the plant part and its oily extract are simultaneously applied, or the essential oil is externally applied (4 citations, 1.1%, 3 citations, 0.8%, 1 citation, 0.3% respectively). The most common preparation forms are demonstrated in Figure 5. In certain cases, plant parts are crushed, heated, boiled, or dried, whilst they can be applied fresh directly on the affected area. The local people utilize also additional ingredients for the preparation of herbal recipes, such as olive or sesame oil, milk, wine, soap, egg white, tomato pulp, sugar, whitewash, vinegar, flour, “tsipouro” which is an alcoholic distilled spirit, and wax. Most of them represent the common ingredients, frequently employed in the history of medicine for the preparation of concoctions used against various dermatological ailments. Though, in the present study, some noteworthy medications are mentioned and described. One of the most distinct herbal remedies is prepared using the bulbs of *A. sativum*, which are smashed and mixed with soap, egg white and “tsipouro” (alcohol), to create a paste called “blathri”. “Blathri” is topically applied on the skin to cure oedema and skin inflammations. The term “blathri” or “blástri” (Greek έμπλαστρον) means plaster or cataplasm. More specifically it refers to a medicinal preparation (cloth coated with medicinal substances), which is applied to diseased parts of the body on the skin, usually to relieve pain (Babiniotis, 2019). In the study area, among the most popular recipes applied for the healing of aphthae and mouth sores is the preparation of “madzúni”. It is worth mentioning here that the word “madzúni”, “mandzúni”, or “mantzuni” (Turkish macun, Greek μα(ν)τζούνι), is widely used in Greek folk medicine referring to any herbal preparation (usually with honey) for the treatment of several ailments and is not specific to a plant species (Ünüsan, 2019). The preparation mentioned in the studied area is composed of the fruits of *M. nigra*, boiled with sugar to create a thick solution stored in the fridge of every house and is widely used (Figure 6). In rare circumstances, medicinal plants are internally used to treat skin illnesses. However, local people utilize bulbs of *A. coronaria* crushed and mixed with flour, to prepare pills administered as medication against haemorrhoids. Additionally, in regard to the information provided on the conservation of some medication forms, it is interesting to highlight that *M. nigra* solution is stored in the fridge after its preparation and *H*. *perforatum* oil is reported to be prepared in two different ways. In some cases, after the immersion of the inflorescences in olive oil, the bottle is exposed to sunlight for 40 days, while in other cases the bottle is conserved in the dark for the same period of time.

### 3.5 Quantitative analysis

#### 3.5.1 Informant Consensus Factor (FIC)

The skin ailments cited were categorized into 18 different groups. As demonstrated in Table 1, FIC was calculated for all ailment categories. High value of FIC indicates the agreement of selection of plant species between informants, whereas a low value indicates disagreement. The agreement ratio analysis has been frequently used as an important tool for the analysis of ethnobotanical data (Uprety et al., 2010; Uniyal et al., 2011). In the present study, the FIC values ranged from 0 to 1. The highest values are observed for Freckles (1) and Haemostatic (1). The species mentioned to be employed for these skin problems are *Vitis vinifera* L. (bark’s juice topically applied) and *F. ornus* (fresh leaves topically applied) respectively. In fact, in these cases the informants that mentioned the use of *V. vinifera* and *F. ornus* agreed on their use exclusively against freckles and as haemostatic respectively. Aphthae and mouth sores were recorded to have the second highest FIC (0.96). *M. nigra* represents the most cited plant remedy indicated for this use. Irritations were ranked as the third ailment group with a FIC value of 0.92 and eczema was rated as the fourth (0.91).

#### 3.5.2 Fidelity level (FL)

The fidelity level value is an important means to estimate for which ailment a particular species is more effective (Chaachouay et al., 2019). In our study, FL was calculated for the most relevant species cited at least 8 times by the informants and varied from 2% to 100%. The analysis revealed 7 species with a FL of 100% (Table 2), most of which were used in a single ailment category and mentioned by multiple informants. More specifically, the highest FL of 100% was recorded for *F. ornus* (Cuts, Wounds, Burns, Ulcers), *H. spectabile* (Calluses), *H*. *perforatum* (Cuts, Wounds, Burns, Ulcers), *M. nigra* (Aphthae, Mouth sores), *Plantago* sp. (Cuts, Wounds, Burns, Ulcers), *P. avium* (Cuts, Wounds, Burns, Ulcers), *Q. coccifera* (Eczema). In the main, a FL of 100% for a particular plant demonstrated that all the reports referred to the same therapeutic use. Other species characterized by a high FL value is *A. cepa* 91% (Bruises, Contusions, Oedemas) and *M. sylvestris* 83% (Irritations and Insect stings, Animal bites). The taxa highly cited should be taken into further consideration for the development of healing agents. Besides, the plant species with low FL% should also be considered and communicated to future generations, to avoid the extinction of ethnobotanical knowledge.

#### 3.5.3 Use Value (UV)

The UV index helps to identify the most important plant species within a population or culture. Based on the results obtained from its calculation for all plant species documented in the study, it was proved that its value ranged between 0.02 and 1.17 (Table 3). According to UV analysis, the species with the highest UV are *H. perforatum* (1.17), *Plantago* sp*.* (0.97), *Q. coccifera* (0.42), *M. chamomilla* (0.38), *M. nigra* (0.35), *M. sylvestris* (0.33), *A. cepa* (0.32), *A. sativum* (0.32), *F. ornus* (0.22), and *H. spectabile* (0.17). The medicinal plants characterized by a high UV were mentioned by a high number of informants. An example is *H*. *perforatum* characterized by the highest UV (1.17) and mentioned by the highest number of informants (70 citations by 48 informants). This indicates the significance of these species for the medication of skin pathological conditions and skin care in the study area. In the present study, species with a low UV (0.02) and mentioned by only one informant were also recorded. Among these are *U. dioica*, *Thymus* sp., *S. melongena*, and *Olea europaea* L.

## 4 Discussion

Nowadays, the development of ethnopharmacological fieldwork results in an increased collection of information relative to the medicinal uses of plants. This prompts pharmacognosy and chemistry of natural products to evaluate the medicinal properties and pharmaceutical potential of documented medicinal flora through the exploration of plant species, and specifically through the isolation, identification and biological evaluation of their secondary metabolites. The ethnopharmacological approach toward the assessment and appraisal of traditional and herbal medicinal practices promotes the involvement of both social and natural sciences (Leonti and Casu, 2013). Despite the large number of ethnopharmacological surveys in Europe (Quave et al., 2012) and the Balkans (Jarić et al., 2007; Menković et al., 2011; Šarić-Kundalić et al., 2011; Pieroni et al., 2015; Živković et al., 2020), Greece remains insufficiently investigated from this point of view. In addition, none of the studies carried out in Greece has documented the uses of medicinal plants exclusively against skin disorders. According to the World Health Organization, 80% of the world’s population uses herbal medicines to treat a significant number of diseases, including skin diseases (Farnsworth et al., 1987; World Health Organization, 2013). Therefore, the present study emphasizes on the uses of medicinal plants for the treatment of skin ailments, on Mount Pelion, an area not adequately studied to date, but with relevant biodiversity and a variety of medicinal plants. Mount Pelion is an alpine Greek area historically exposed to very few external influences and centered around a subsistence economy. Therefore, it represents a study area where the cultural extinction is less notable and the conservation of plant biodiversity is significant. This documentation contributes to the preservation of both cultural, social and economic identity and traditional ethnobotanical knowledge in Greece. The elaboration of the data demonstrated an important necessity to conduct more ethnopharmacological research in the study area as well as in other regions in Greece. Traditional medical practices should additionally be evidenced through the consultation of ancient manuscripts. Most of the cited species have ethnopharmacological relevance for the treatment of skin problems either due to their direct healing effects or because of their anti-inflammatory and antibacterial activity. The results of the present survey revealed that the employment of medicinal species in a population, as well as the preservation of indigenous ethnopharmacological knowledge, are culturally significant and could empower future research and promote ethnopharmacological advances. Additionally, natural products are obtaining a protagonist role in skin healing procedures, such as the multifunctional and complex process of wound healing pathophysiology. Among the factors that increase scientific interest regarding natural product uses are their multitargeted biological activities (Gertsch, 2011). The exploitation of traditional therapeutic information combined with the study of the biological activity of natural products creates a pole of scientific interest for the design of healing preparations. The ultimate goal is the utilization of ethnopharmacological data for the discovery of new bioactive natural products and promising compounds against skin diseases.

## Data Availability

The original contributions presented in the study are included in the article/Supplementary material, further inquiries can be directed to the corresponding authors.

## References

[B1] Axiotis E. Halabalaki M. Skaltsounis L. A. (2018). An ethnobotanical study of medicinal plants in the Greek islands of North Aegean region. Front. Pharmacol. 9, 409. 10.3389/fphar.2018.00409 29875656 PMC5974156

[B2] Babiniotis G. (2019). Λεξικό της νέας ελληνικής γλώσσας: Με σχόλια για τη σωστή χρήση των λέξεων. Athens: Kéntro Lexikologías.

[B3] Brussell D. (2004). Medicinal plants of Mt. Pelion, Greece. Econ. Bot. 58, S174–S202. 10.1663/0013-0001(2004)58[s174:mpompg]2.0.co;2

[B4] Chaachouay N. Benkhnigue O. Fadli M. El Ibaoui H. Zidane L. (2019). Ethnobotanical and ethnopharmacological studies of medicinal and aromatic plants used in the treatment of metabolic diseases in the Moroccan Rif. Heliyon 5, e02191. 10.1016/j.heliyon.2019.e02191 31720440 PMC6838988

[B5] Chourmouziadis G. Asimakopoulou-Atzaka P. Makris K. A. (1982). Μαγνησία: Το χρονικό ενός πολιτισμού. Athens: Kapon.

[B6] Danna C. Poggio L. Smeriglio A. Mariotti M. Cornara L. (2022). Ethnomedicinal and ethnobotanical survey in the aosta valley side of the gran paradiso national park (western alps, Italy). Plants 11, 170. 10.3390/plants11020170 35050058 PMC8778718

[B7] Dimopoulos P. Raus T. Bergmeier E. Constantinidis T. Iatrou G. Kokkini S. (2016). Vascular plants of Greece: An annotated checklist. Supplement. Willdenowia 46, 301–347. 10.3372/wi.46.46303

[B8] Doyle B. J. Asiala C. M. Fernández D. M. (2017). Relative importance and knowledge distribution of medicinal plants in a kichwa community in the Ecuadorian amazon. Ethnobiol. Lett. 8. 10.14237/ebl.8.1.2017.777

[B9] Farnsworth N. Akerele O. Bingel A. (1987). Medicinal plants in therapy. J. Ethnopharmacol. 19, 336. 10.1016/0378-8741(87)90016-x PMC2536466 3879679

[B10] Flora of Greece web Vascular Plants of Greece An annotated Checklist (2023). Flora Greece web vasc. Plants Greece annot. Checkl. Available at: https://portal.cybertaxonomy.org/flora-greece/intro (Accessed October 7, 2023).

[B11] Friedman J. Yaniv Z. Dafni A. Palewitch D. (1986). A preliminary classification of the healing potential of medicinal plants, based on a rational analysis of an ethnopharmacological field survey among Bedouins in the Negev Desert, Israel. J. Ethnopharmacol. 16, 275–287. 10.1016/0378-8741(86)90094-2 3747566

[B12] Gertsch J. (2011). Botanical drugs, synergy, and network Pharmacology: Forth and back to intelligent mixtures. Planta Med. 77, 1086–1098. 10.1055/s-0030-1270904 21412698

[B13] Global Biodiversity Information Facility (2022). Glob. Biodivers. Inf. Facil. Available at: https://www.gbif.org/ (Accessed May 2, 2023).

[B14] Hanlidou E. Karousou R. Kleftoyanni V. Kokkini S. (2004). The herbal market of Thessaloniki (N Greece) and its relation to the ethnobotanical tradition. J. Ethnopharmacol. 91, 281–299. 10.1016/j.jep.2004.01.007 15120452

[B15] Hay R. J. Johns N. E. Williams H. C. Bolliger I. W. Dellavalle R. P. Margolis D. J. (2014). The global burden of skin disease in 2010: An analysis of the prevalence and impact of skin conditions. J. Invest. Dermatol. 134, 1527–1534. 10.1038/jid.2013.446 24166134

[B16] Heinrich M. (2000). Ethnobotany and its role in drug development. Phytother. Res. 14, 479–488. 10.1002/1099-1573(200011)14:7<479:AID-PTR958>3.0.CO;2-2 11054835

[B17] Jarić S. Popović Z. Mačukanović-Jocić M. Djurdjević L. Mijatović M. Karadžić B. (2007). An ethnobotanical study on the usage of wild medicinal herbs from Kopaonik Mountain (Central Serbia). J. Ethnopharmacol. 111, 160–175. 10.1016/j.jep.2006.11.007 17145148

[B18] Karousou R. Balta M. Hanlidou E. Kokkini S. (2007). "Mints", smells and traditional uses in Thessaloniki (Greece) and other Mediterranean countries. smells traditional uses Thessalon. (Greece) other Mediterr. Ctries. *J. Ethnopharmacol.* 109, 248–257. 10.1016/j.jep.2006.07.022 16962274

[B19] Kleftoyanni V. Kokkini S. (2003). The Labiatae plants used traditionally in Thessaloniki. Bocconea 16, 1117–1121.

[B20] Kougioumoutzis K. Kokkoris I. Panitsa M. Kallimanis A. Strid A. Dimopoulos P. (2021). Plant endemism centres and biodiversity hotspots in Greece. Biology 10, 72. 10.3390/biology10020072 33498512 PMC7909545

[B21] Leonti M. Casu L. (2013). Traditional medicines and globalization: Current and future perspectives in ethnopharmacology. Front. Pharmacol. 4, 92. 10.3389/fphar.2013.00092 23898296 PMC3722488

[B22] Lietava J. (1992). Medicinal plants in a middle paleolithic grave shanidar IV? J. Ethnopharmacol. 35, 263–266. 10.1016/0378-8741(92)90023-K 1548898

[B23] Malamas M. Marselos M. (1992). The tradition of medicinal plants in Zagori, Epirus (northwestern Greece). J. Ethnopharmacol. 37, 197–203. 10.1016/0378-8741(92)90034-O 1453708

[B24] Menković N. Šavikin K. Tasić S. Zdunić G. Stešević D. Milosavljević S. (2011). Ethnobotanical study on traditional uses of wild medicinal plants in Prokletije Mountains (Montenegro). J. Ethnopharmacol. 133, 97–107. 10.1016/j.jep.2010.09.008 20837123

[B25] Moustris K. P. Proias G. T. Larissi I. K. Nastos P. T. Koukouletsos K. V. Paliatsos A. G. (2016). Health impacts due to particulate air pollution in Volos City, Greece. J. Environ. Sci. Health Part A 51, 15–20. 10.1080/10934529.2015.1079099 26421944

[B26] Niavis S. Tamvakis N. Manos B. Vlontzos G. (2018). Assessing and explaining the efficiency of extensive olive oil farmers: The case of pelion peninsula in Greece. Agriculture 8, 25. 10.3390/agriculture8020025

[B27] Papageorgiou D. Bebeli P. J. Panitsa M. Schunko C. (2020). Local knowledge about sustainable harvesting and availability of wild medicinal plant species in Lemnos island, Greece. J. Ethnobiol. Ethnomedicine 16, 36. 10.1186/s13002-020-00390-4 PMC7304145 32560660

[B28] Papanastasiou D. K. Melas D. (2009). Climatology and impact on air quality of sea breeze in an urban coastal environment. Int. J. Climatol. 29, 305–315. 10.1002/joc.1707

[B29] Papathanasiou A. D. (2006). Το χρονικό του aγίου ?αυρεντίου 14ος-19ος αιώνες. Thessaly: Institutional Repository - Library and Information Centre - University of Thessaly.

[B30] Perouli M. Bareka P. (2022). Ethnobotanical survey on medicinal plants from Milos island (Kiklades Islands, Greece). Mediterr. Bot. 43, e75357. 10.5209/mbot.75357

[B31] Petrakou K. Iatrou G. Lamari F. N. (2020). Ethnopharmacological survey of medicinal plants traded in herbal markets in the Peloponnisos, Greece. J. Herb. Med. 19, 100305. 10.1016/j.hermed.2019.100305

[B32] Petrovska B. (2012). Historical review of medicinal plants′ usage. Pharmacogn. Rev. 6, 1–5. 10.4103/0973-7847.95849 22654398 PMC3358962

[B33] Phillips O. Gentry A. H. Reynel C. Wilkin P. Galvez-Durand B, C. (1994). Quantitative ethnobotany and amazonian conservation. Conserv. Biol. 8, 225–248. 10.1046/j.1523-1739.1994.08010225.x

[B34] Pieroni A. Ibraliu A. Abbasi A. M. Papajani-Toska V. (2015). An ethnobotanical study among Albanians and Aromanians living in the Rraicë and Mokra areas of Eastern Albania. Genet. Resour. Crop Evol. 62, 477–500. 10.1007/s10722-014-0174-6

[B35] Posnett J. Gottrup F. Lundgren H. Saal G. (2009). The resource impact of wounds on health-care providers in Europe. J. Wound Care 18, 154–161. 10.12968/jowc.2009.18.4.41607 19349935

[B36] Quave C. L. Pardo-de-Santayana M. Pieroni A. (2012). Medical ethnobotany in Europe: From field ethnography to a more culturally sensitive evidence-based CAM? Evid. Based Complement. Altern. Med. 2012, 156846–156917. 10.1155/2012/156846 PMC3413992 22899952

[B37] Rossato S. C. Leitao-Filho H. D. F. Begossi A. (1999). Ethnobotany of caicaras of the atlantic forest coast (Brazil). Econ. Bot. 53, 387–395. 10.1007/BF02866716

[B38] Šarić-Kundalić B. Dobeš C. Klatte-Asselmeyer V. Saukel J. (2011). Ethnobotanical survey of traditionally used plants in human therapy of east, north and north-east Bosnia and Herzegovina. J. Ethnopharmacol. 133, 1051–1076. 10.1016/j.jep.2010.11.033 21094241

[B39] Seth D. Cheldize K. Brown D. Freeman E. E. (2017). Global burden of skin disease: Inequities and innovations. Curr. Dermatol. Rep. 6, 204–210. 10.1007/s13671-017-0192-7 29226027 PMC5718374

[B40] Singh A. G. Kumar A. Tewari D. D. (2012). An ethnobotanical survey of medicinal plants used in Terai forest of Western Nepal. J. Ethnobiol. Ethnomedicine 8, 19. 10.1186/1746-4269-8-19 PMC3473258 22591592

[B41] Skoula M. D’Agata C. D. C. Sarpaki A. (2009). Contribution to the ethnobotany of Crete, Greece. Bocconea 23, 479–487.

[B42] Trotter R. T. Logan M. H. (1986). “Informant consensus: A new approach for identifying potentially effective medicinal plants,” in Plants and indigenous medicine and diet (New York: Routledge), 22.

[B43] Tsioutsiou E. E. Amountzias V. Vontzalidou A. Dina E. Stevanović Z. D. Cheilari A. (2022). Medicinal plants used traditionally for skin related problems in the south Balkan and east Mediterranean region—a review. Front. Pharmacol. 13, 936047. 10.3389/fphar.2022.936047 35865952 PMC9294246

[B44] Tsioutsiou E. E. Giordani P. Hanlidou E. Biagi M. De Feo V. Cornara L. (2019). Ethnobotanical study of medicinal plants used in Central Macedonia, Greece. Evid. Based Complement. Altern. Med. 2019, 4513792–4513822. 10.1155/2019/4513792 PMC6463668 31057648

[B45] Uniyal S. Kr. Sharma V. Jamwal P. (2011). Folk medicinal practices in kangra district of Himachal Pradesh, western himalaya. Hum. Ecol. 39, 479–488. 10.1007/s10745-011-9396-9

[B46] Ünüsan N. (2019). Systematic review of mycotoxins in food and feeds in Turkey. Food control. 97, 1–14. 10.1016/j.foodcont.2018.10.015

[B47] Uprety Y. Asselin H. Boon E. K. Yadav S. Shrestha K. K. (2010). Indigenous use and bio-efficacy of medicinal plants in the Rasuwa District, Central Nepal. J. Ethnobiol. Ethnomedicine 6, 3. 10.1186/1746-4269-6-3 PMC2823594 20102631

[B48] Vamvakos S. (1927). Ιστορία του χωριού Άγιος Λαυρέντιος του Βόλου. Από αρχαιοτάτων χρόνων μέχρι σήμερον.pdf. Athens: Municipality of Agios Lavrentios.

[B49] Vokou D. Katradi K. Kokkini S. (1993). Ethnobotanical survey of Zagori (Epirus, Greece), a renowned centre of folk medicine in the past. J. Ethnopharmacol. 39, 187–196. 10.1016/0378-8741(93)90035-4 8258976

[B50] World Flora Online Plant List (2022). World flora online plant list. Available at: https://wfoplantlist.org/plant-list (Accessed January 31, 2023).

[B51] World Health Organization (2013). WHO traditional medicine strategy: 2014-2023. Geneva: World Health Organization. Available at: https://apps.who.int/iris/handle/10665/92455 (Accessed January 31, 2023).

[B52] Zenderland J. Hart R. Bussmann R. W. Paniagua Zambrana N. Y. Sikharulidze S. Kikvidze Z. (2019). The use of “use value”: Quantifying importance in ethnobotany. Econ. Bot. 73, 293–303. 10.1007/s12231-019-09480-1

[B53] Živković J. Ilić M. Šavikin K. Zdunić G. Ilić A. Stojković D. (2020). Traditional use of medicinal plants in south-eastern Serbia (pčinja district): Ethnopharmacological investigation on the current status and comparison with half a century old data. Front. Pharmacol. 11, 1020. 10.3389/fphar.2020.01020 32733251 PMC7360817

